# Optofluidic UV-Vis spectrophotometer for online monitoring of photocatalytic reactions

**DOI:** 10.1038/srep28928

**Published:** 2016-06-29

**Authors:** Ning Wang, Furui Tan, Yu Zhao, Chi Chung Tsoi, Xudong Fan, Weixing Yu, Xuming Zhang

**Affiliations:** 1 The Hong Kong Polytechnic University Shenzhen Research Institute, Shenzhen, P.R. China; 2 Department of Applied Physics, The Hong Kong Polytechnic University, Hong Kong, P.R. China; 3 Institute of Functional Nano & Soft Materials (FUNSOM) & Collaborative Innovation Center of Suzhou Nano Science and Technology, Jiangsu Key Laboratory for Carbon-Based Functional Materials & Devices, Soochow University, Suzhou, Jiangsu, P.R. China; 4 Department of Biomedical Engineering, University of Michigan, Ann Arbor, MI 48109, USA; 5 Key Laboratory of Spectral Imaging Technology, Xi’an Institute of Optics and Precision Mechanics, Chinese Academy of Sciences, Xi’an, Shaanxi, P.R. China

## Abstract

On-chip integration of optical detection units into the microfluidic systems for online monitoring is highly desirable for many applications and is also well in line with the spirit of optofluidics technology–fusion of optics and microfluidics for advanced functionalities. This paper reports the construction of a UV-Vis spectrophotometer on a microreactor, and demonstrates the online monitoring of the photocatalytic degradations of methylene blue and methyl orange under different flow rates and different pH values by detecting the intensity change and/or the peak shift. The integrated device consists of a TiO_2_-coated glass substrate, a PDMS micro-sized reaction chamber and two flow cells. By comparing with the results of commercial equipment, we have found that the measuring range and the sensitivity are acceptable, especially when the transmittance is in the range of 0.01–0.9. This integrated optofluidic device can significantly cut down the test time and the sample volume, and would provide a versatile platform for real-time characterization of photochemical performance. Moreover, its online monitoring capability may enable to access the usually hidden information in biochemical reactions like intermediate products, time-dependent processes and reaction kinetics.

Optical detection systems for optofluidic devices are often macroscopic, expensive and inflexible, and usually requires sophisticated optical design and coordination of light sources, detectors and other subunits^1^,^2^,^3^,^4^. In addition, reconfiguration of the optical detection systems is time-consuming and inconvenient, and thus inefficient for online monitoring. Meanwhile, for some microfluidic devices, the assembly process is complicated and costly, making it challenging to conduct experimental operation and scaling-up fabrication^5^,^6^,^7^,^8^,^9^,^10^. Therefore, it is highly desirable to develop integrated, simple yet efficient optical detection system in the optofluidic chips to overcome the existing challenges.

One of the very popular optical instruments is the UV-visible (UV-Vis) spectrophotometer, which is extensively used to measure the absorption spectrum of samples in the UV and visible regions. In our previous work^11^,^12^,^13^,^14^,^15^, we demonstrated photocatalytic planar microreactors (typical dimensions 5 cm × 1.8 cm × 100 μm) with much enhanced efficiencies of mass transfer and photon transfer for photocatalysis water purification. Nevertheless, large amount of stock solutions is needed in order to measure the optical absorbance using the UV-Vis spectrophotometer (UV-2550, Shimadzu). This is because the photocatalytic microreactor cannot be inserted directly into the optical path of the bulky UV-Vis spectrophotometer due to the space limit and the incompatibility of optical subsystems. The sample solutions have to be collected into quartz cuvettes before they are put into the UV-Vis spectrophotometer for spectral measurement. Each measurement requires a sample volume of at least 3 mL to fill the 1-cm cuvette, while one run of test usually needs 20–200 measurements (to try different conditions and to repeat the tests). This makes the measurements time-consuming and offsets the major merits of microreactors (e.g., fast reaction speed, save of the solution volume). Moreover, the sample solutions are usually sensitive to environment changes, and thus the process of first collection and then measurement complicates the experimental operations and brings errors. To solve the problems, it is necessary to integrate the optical detection system into the optofluidic devices to enable the online and real-time monitoring of the reaction processes^16^. Take the photodegradation as an example, the integrated monitor system can simplify the complicated optical configuration and the experimental operation. Methylene blue (MB) has been investigated as the most popular reagent for photocatalytic degradation studies. In short, the principle of degradation is that the chromophore of MB molecules is broken by photon-excited redox, accompanied with complex oxidation and reduction reactions, including mineralization of carbon, nitrogen and sulfur heteroatoms into CO_2_, NH_4_^+^, NO_3_^−^ and SO_4_^2−^, respectively^17^. However, the degradation pathway is not simplex and there might be intermediate products in different reaction environments. For instance, aromatics experiences successive hydroxylations, which lead to the aromatic ring opening. Therefore, the integrated system is of great significance for real-time monitoring of the contents of some aromatics materials. This may be envisaged as a quick tool to study the dye degradation pathways and to further examines the detailed mechanisms of MB photodegradation.

In this work, we will construct a UV-Vis spectrophotometer integrated onto a photocatalytic microreactor as the online monitoring module and will demonstrate its functionalities through real-time detection of the photochemical reactions. Two detection modes will be examined: *intensity-based monitoring* and *spectrum-based monitoring*. For the former, the MB is used as the model chemical since its characteristic absorption peak is always fixed at around 664 nm. In this case, a laser is used as the light source for the online monitoring module, and the concentration change can be determined by measuring the intensity change of transmittance at 664 nm. For the latter, methyl orange (MO) is used as the model since its characteristic absorption peak is shifted with the pH value and the photoreaction process. And a broadband light source is necessary to trace the wavelength shifts and the intensity changes of absorption peaks. These two detection modes are suitable for most of the biomedical and chemical analyses and the successful demonstration will showcase the versatility of the on-chip UV-Vis spectrophotometer for integration with other microfluidic systems.

## Design and Experiment

### Setup of microreactor system

**Figure 1:**
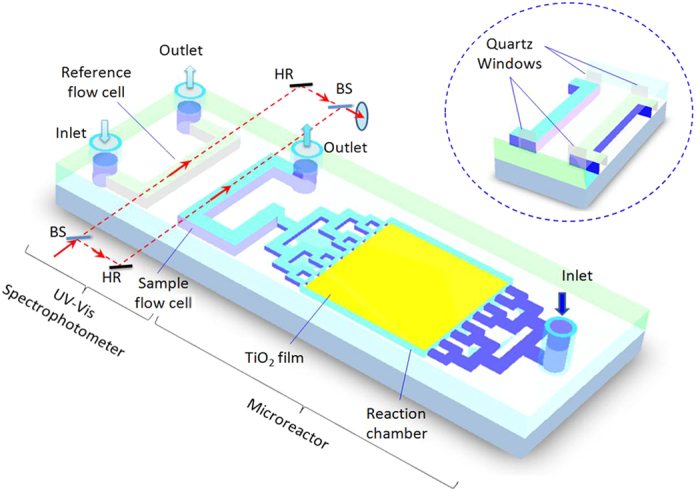
Schematic diagram of the optofluidic device that has a photocatalytic microreactor and an on-chip integrated UV-Vis spectrophotometer. The inset shows the quartz windows inserted between the fluid and the PDMS sidewalls to obtain the smooth interfaces for maximum light transmittance. BS: beam splitter; HR: high-reflection mirror.

**Figure 2:**
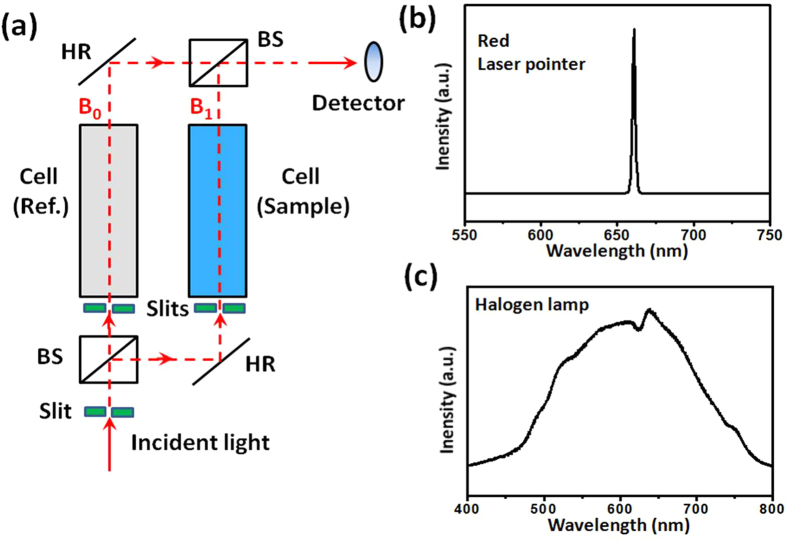
(**a**) Optical configuration of the on-chip UV-Vis spectrophotometer; (**b**) calibrated emission spectrum of the red laser pointer to be used as the monochromic light source; (**c**) calibrated emission spectrum of the halogen lamp as the broadband light source. In (**a**), BS: beam splitter; HR: high-reflection mirror.

Figure 1 schematically illustrates the setup of optofluidic device, which has basically a PDMS structural layer sealed onto a glass substrate (7.5 cm × 2.5 cm). The whole device has two functional parts: *photocatalytic microreactor* and *UV-Vis spectrophotometer*. The microreactor consists of two dendritic microchannels for fluid input/output and a reaction chamber (10 mm × 10 mm × 0.1 mm, total volume 10 μL). The region of the glass substrate right beneath the reaction chamber is coated with a thin layer of photocatalytic TiO_2_ film (10 mm × 10 mm, thickness 2 μm), the same dimension with the reaction chamber. The dendritic microchannels ensure the fluid to flow evenly through the reaction chamber so as to obtain maximum contact with the TiO_2_ film. The on-chip spectrophotometer consists of two flow cells (0.2 mm × 0.2 mm × 10 mm, volume 400 nL) integrated on the same chip of microreactor. One cell is for sample and the other is for reference. As shown more clearly in Fig. 2a, the spectrophotometer has some external components such as slits, a light source, two beam splitters, two mirrors and a detector. The slits here are to control the size and the intensity of the incident light and to prevent the stray light from entering the flow cells. Otherwise, the intensity noise level rises up and the spectral resolution goes down. It is seen that the optical input and output rely on free-space coupling, which is different from the reported studies that embedded optical fibers on the chips for optical coupling^18^. We have tried both methods and have found that the free-space coupling is more stable and convenient. This is because the PDMS material used for the microreactor is soft, making it difficult to maintain the precise optical alignment between the fibers during the operation.

### Materials and methods

All chemicals were of analytical reagent grade and were purchased from Sigma-Aldrich Co., including MB reagent, MO powder, polyethylene glycol (PEG 20000), detergent (Triton X-100), sulfuric acid solution and sodium hydroxide powder. Polydimethysiloxane (PDMS, DC184) was purchased as a two-component kit containing the vinyl-terminated base and curing agent from Dow Corning Co. The SU-8 UV-cured optical adhesive was purchased from Microchem Co..

Standard UV lithography was used to fabricate the PDMS microstructure including the reaction chamber and the online monitoring modules, while the plasma bonding method was used to fabricate the integrated device. A commercial UV-Vis spectrophotometer (UV-2550, Shimadzu) was used to calibrate the concentration change between the original and the degraded MB solution. A 660-nm laser pointer and a halogen lamp (EKE 21V, 150 W) were used as the monochromatic and broadband light sources to monitor the MB and MO degradation, respectively. A hand-held UV lamp with two modulator tubes (rated output power 16 W) is used as the light source for photodegradation.

### Fabrication of integrated optofluidic device

First, the TiO_2_ photocatalytic film was fabricated. The TiO_2_ porous film with large surface area was prepared by the manual painting method developed in our previous work^13^. Details of the fabrication are described in the supplementary material. The as-prepared TiO_2_ thin film exhibited sub-micron porous structure and good homogeneity with a thickness of approximately 2 μm. Next, the reaction chamber and flow cells were fabricated. The reaction chamber and flow cells were replicated using PDMS from a SU-8 mold, which was patterned by standard UV photolithography. The specific method and process are also illustrated in supplementary material.

It should be noted that additional steps were performed to make all the air/PDMS and PDMS/liquid interfaces optically flat, especially those in the flow cells that intersect the optical paths. This is to reduce the scattering and thus the optical loss. For the PDMS/liquid interfaces inside the flow cells, two pieces of quartz sheets (100 μm thick) were adhered to the SU-8 mold parts for the flow cells. After the PDMS replication, the quartz sheets form the inner surfaces of the flow cells. For the air/PDMS interfaces, a thin layer of uncured PDMS was first poured on a silicon wafer; then the rough PDMS sidewalls of the already-bonded chip were pressed against the uncured PDMS layer, followed by a curing process at 70 °C for 30 min. After the curing, the bonded chip was peeled off from the wafer. Now, the initially very rough air/PDMS interfaces became optically clear and flat^19^.

### Calibration of integrated device

First, the light source was calibrated. The light intensity of the monochromatic laser pointer was measured by an optical power meter (2636, Newport, photodetector: 918D-UV-OD3) and the emission wavelength was measured by an integrating sphere (Labsphere). A single peak was observed at around 660 nm with a narrow linewidth of 20 nm as shown in Fig. 2b. The emission spectrum of the halogen lamp were measured by an optical spectrometer (Ocean Optics, 4000), showing a broad emission wavelength range of 450–750 nm as shown in Fig. 2c. The variation of light intensity was measured to be below 5% in 2 hours. These data were used as the standard for further experiments.

Then, the concentration of solution was calibrated. Different reagents with standard concentration (0.05% wt.) were prepared. During the testing process, the intensity of transmitted light should be kept low, usually in the order of micro-watts, to avoid excessive light scattering. The slit was used to adjust the spot size and the intensity of light.

### Online monitoring of photodegradation by the device

In the experiments of photocatalytic degradation, the MB and MO solutions were used as the model chemicals. The initial concentration of both solutions was 3 × 10^−5^ mol/L. The original reagent was introduced into the microreactor by a syringe pump (TS2-60, Longer). The photodegraded solution flowing through the flow cells were measured by the on-chip UV-Vis spectrophotometer. For comparison, the photodegraded solution of 3 mL was collected from the outlet and then measured by the commercial UV-Vis spectrophotometer. Detailed tests were conducted to determine the influences of various parameters of the initial solutions, such as flow rates (controlled by the syringe pump) and pH values (adjusted by adding NaOH or H_2_SO_4_ solutions).

## Experimental results

### Calibration of MB solutions with testing modules

The Beer-Lambert law^20^,^21^ is employed to calculate the absorbance, which is given by,


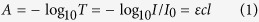


where *A* is absorbance, *T* is transmittance, *I* and *I*_0_ are the light intensity of incident light and transmittance light, respectively, *ε* is the molar absorptivity with units of L mol^−1^ cm^−1^, *c* is the concentration of the compound in solution, expressed in mol L^−1^, *l* is the path length of the sample in the flow cells.

**Figure 3:**
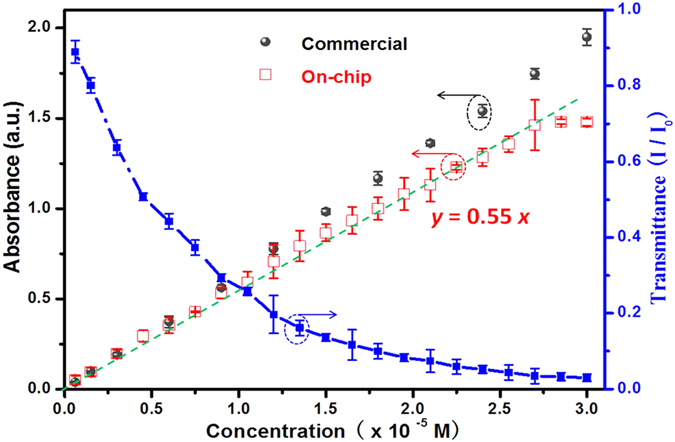
Calibration of the concentration measurement using the standard methylene blue solution samples. The black round points represent the absorption results given by the commercial UV-Vis spectrophotometer, while the red half hollow square points by our device. The corresponding transmittance is also plotted using the blue solid square points. a.u. denotes arbitrary unit. *I* is the transmitted intensity, and *I*_0_ is the incident intensity.

To calibrate the performance of the on-chip UV-Vis spectrophotometer, the original concentration of the MB aqueous solution was diluted to 20 different concentrations ranging from 2% to 95% by adding DI water. The absorbance and transmittance versus the concentration are plotted in Fig. 3. Each data point was repeated for four times. Compared with the standard results obtained by the commercial UV-Vis spectrophotometer, our measurement data approach approximately the standard results, though smaller. The difference becomes obvious with the increase of the concentration, especially in the high concentration range (>2.7 × 10^−5^ mol/L). And, the error bars of our data are wider than the standard results. The imperfection of our on-chip UV-Vis spectrophotometer may be attributed to the non-monochromaticity of the laser point light, the fabrication error, the concentration inaccuracy and the difference of test conditions (e.g., ambient light background, temperature). Nevertheless, our data points with the concentration below 90% maintain a good linearity and can be well fitted to the expression


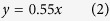


here *y* is the absorbance and *x* is the concentration (in the unit of 10^−5^ mol/L). This suggests that our device is suitable for the measurements of low concentrations.

Beyond that, we found that the two points at 2.7 × 10^−5^ M and 3 × 10^−5^ M fall off the fit and seem to be saturated. That is because the solution with high MB in the sample cell absorbs most of the light, causing the weak transmitted light to be comparable to the noise level of the detector. As a result, the upper limit of the detection of the on-chip device is 2.7 × 10^−5^ M for MB solutions. For the lower limit of the on-chip device, it is close to the commercial one, normally 10^−7^ M.

### Online monitoring of MB degradation

In the microreactor system, the flow rate is one of the major factors that affect the photocatalytic reaction efficiency. To investigate this effect, the solutions were pumped into the reaction chamber and flow cells at the flow rate of 37.5, 50, 75 and 150 μL/min, which correspond to the residence times of 16, 12, 8 and 4 s, respectively. Here, the residence time means the time for the MB solution to flow through the reaction chamber, which also can be defined as reaction time and expressed by the following relationship: Residence time = Reaction time = Volume of reaction chamber/Flow rate.

**Figure 4:**
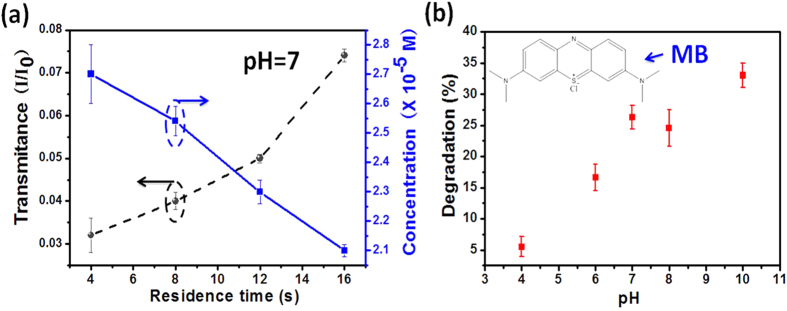
Online measurements of the photocatalytic efficiency in decomposing the methylene blue. (**a**) Different flow rates at pH = 7; (**b**) different pH values at the residence time of 12 s (corresponding to the flow rate of 50 μL/min). The inset shows the molecular structure of methylene blue.

Figure 4a plots the concentration changes with respect to the residence time at pH 7, shown by the solid line. The corresponding transmittances under different flow rates are plotted by the dash line relative to the calibration data points in Fig. 3. The data points are the average values of 4 tests. Here, the testing results could be roughly obtained by the fitted expression Eq. (2).

Figure 4b shows the degradation of MB under different initial pH values measured by the on-chip UV-Vis spectrophotometer. The flow rate was fixed at 50 μL/min. At different pH values, the absorption peak of MB solution was always centered at about 660 nm. It can be seen that the degradation efficiency increases with the pH value. This trend can be explained by the adsorption efficiency of MB dyes on the surface of TiO_2_. It is known that the semiconducting TiO_2_ has amphoteric behavior which means that its surface charge varies with the pH value of the aqueous solution^22^. The transformation of TiO_2_ with acidity can be written as below,


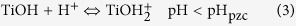



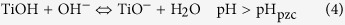


Here, pH_pzc_ represents the pH value of TiO_2_ film at point of zero charge. It can be seen the TiO_2_ surface is positively charged in the acidic condition (low pH value, due to the excessive H^+^ ions) and becomes negatively charged in the alkaline condition (high pH value, due to the excessive OH^−^ ions). Since the MB molecules are positively charged, the electrostatic adsorption to the TiO_2_ surface becomes stronger when the solution is changed from acidic to alkaline, leading to higher degradation efficiency at higher pH value^23^.

### Online monitoring of MO degradation under different pH values

**Figure 5:**
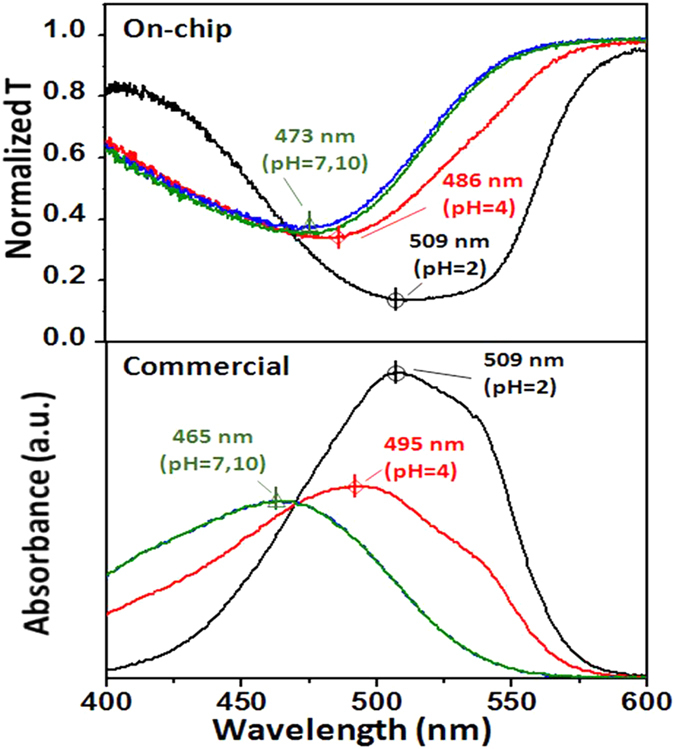
Comparison of the spectral features of methyl orange at different pH values. Upper panel: transmission spectra measured by the on-chip UV-Vis spectrophotometer. Lower panel: absorption spectra measured by the commercial equipment. a.u. denotes arbitrary unit.

To further demonstrate the spectrum-based function by using the broadband light source, the transmission spectra of MO under different pH values were tested by the on-chip UV-Vis spectrophotometer (see the upper panel of Fig. 5) and then compared with the absorbance spectra measured by the commercial equipment (see the lower panel of Fig. 5). Both the absorbance and transmission peak were centered at 509 nm at pH 2. In contrast, the peaks experienced a blue shift at pH = 4 and a red shift at pH = 7, 10 as summarized in Table 1. This is different from the MB molecule whose peaks are independent of the pH value. From Table 1, one can see that the transmission peak positions measured by our on-chip spectrophotometer have small deviation from the absorption peak positions given by the commercial equipment. The maximum shift is 9 nm and the maximum error percentage is <2%. This should be acceptable in consideration of some system errors between the measurement setups.

**Figure 6:**
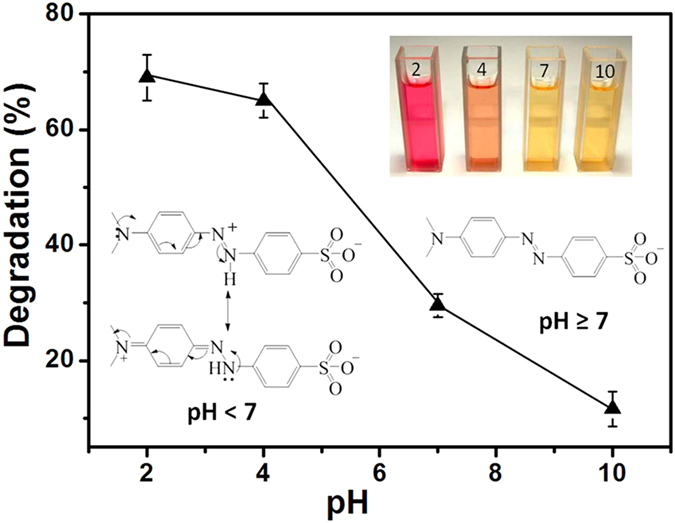
Online measurement of the efficiency decomposing MO under different pH values at the flow rate of 37.5 μL/min. The insets show the colors of solutions and the molecule structures of MO under different pH values that correspond to acidity, neutral & alkalinity.

To examine the influence of pH value on the degradation of MO, photocatalytic reactions were conducted at different pH values. The results are shown in Fig. 6. It is found that the degradation efficiency goes down with the increase of pH value, indicating a slower decomposition rate in the alkaline environment.

The observed influences of the pH value on the absorption/transmission peaks and the degradation efficiency can be explained by considering the fact that the molecule structure of MO would be transformed in response to the change of pH value. The structure of MO molecules is generally shown in Fig. 6 inset when they are dissolved in the acidic solution. When the alkalinity is increased, its molecular structure would be changed into its basic form (Fig. 6 inset), causing a color change from red to orange and finally to yellow (Fig. 6 inset)^24^. Simultaneously, a red shift of the absorption peak also occurs due to the hydrazone structure transforming to its resonance form (Fig. 6 inset). This explains the peak shift in Fig. 5. For the influence of degradation, it is because all the three forms of MO molecules are negatively charged regardless of the acidity. According to Eq. (2), a low pH value leads to a positively charged TiO_2_ surface and thus an enhanced adsorption of MO molecules due to the electrostatic attraction. As a result, the degradation efficiency is high. Oppositely, a high pH value causes to negatively charged TiO_2_ surface, which repels the negatively-charge MO molecules and causes a reduced degradation efficiency. This well explains the data trend in Fig. 6.

## Discussion

This microreactor has a small reaction chamber and two small sample flow cells, which can be used as a rapid test kit to quantify the efficiency of photoreaction and to optimize the operational conditions. In the microreactor, comprehensive information about the ongoing reaction can be obtained as fast as 10 s, which is much superior to other bulk reactors usually taking a few hours. In addition, the on-chip spectrophotometer can be used to detect the intermediate and final products of various chemical reactions in real time, and thus may enable to access the usually-hidden information like the time-dependent compositions, the reaction kinetics and the possible reaction mechanism. Therefore, it is particularly suitable for rapid analysis and characterization of biochemical reactions compared to other *in-situ* sampling and measurement techniques.

As an instrument, good repeatability is a must. In the measurement process of commercial UV-Vis spectrometer, it is very important to clean the cuvettes before each repeated test. In contrast, the online measurement of the on-chip device avoids the complicated process and is thus time-saving. The sample solution can refresh the flow cells by themselves. However, there might be a small amount of MB adsorbed on the inner wall of the flows cells, which may be one of the main factors that affect the measurement precision and the repeatability, especially when the MB concentration is high. Another factor may be the micro-/nano-sized gas bubbles that are often formed in the reaction chamber, which are then subsequently flowed into the flow cells to affect the transmittance by scattering the light. In our experiment, we tried to avoid the bubble formation as much as possible. To improve the measurement repeatability of the on-chip spectrophotometer, the cleaning of the flow cells may be adopted.

Moreover, the external optical setup is adjustable according to real requirement, which is particularly useful if some conditions have been confirmed, such as the targeted chemicals and the corresponding characterized absorption peaks. In addition, the precision and sensitivity of this system might be not comparable with liquid chromatography, but it really omits many complicated and expensive optical elements and can be adjusted according to requirements. In fact, the measuring range and the sensitivity can satisfy most of the requirements, especially in the transmittance range between 0.01 and 0.9.

## Conclusion

An on-chip UV-Vis spectrophotometer for the online monitoring of photocatalytic reactions was constructed by designing and integrating two micro-sized flow cells with a planar reaction chamber. Experimental studies have well demonstrated the functions of the intensity-based detection and the spectrum-based detection by the online monitoring of the photocatalytic degradations of methylene blue and methyl orange under different conditions, and have proven the effectiveness by comparing with the results of commercial equipment. Equipped with the additional merits such as short residence time and small sample volume, the integrated online monitor microreactor system could provide a versatile platform to study the reaction kinetics, to analyze the results in real time, and to reveal the transient processes of photochemical reactions.

## Additional Information

**How to cite this article**: Wang, N. *et al*. Optofluidic UV-Vis spectrophotometer for online monitoring of photocatalytic reactions. *Sci. Rep.* **6**, 28928; doi: 10.1038/srep28928 (2016).

## Supplementary Material

Supplementary Information

## Figures and Tables

**Table 1:** Absorption characteristic peaks *λ* measured by the on-chip UV-Vis spectrophotometer and calibrated by the standard commercial equipment (UV-2550, Shimadzu).

pH	Absorption peak *λ*(nm)		Deviation	
	Measured	Calibrated	Δ*λ*(nm)	%
2	509	509	0	0.0%
4	486	495	9	−1.8%
7	473	465	8	+1.7%
10	473	465	8	+1.7%

The deviation of wavelength Δ*λ* is <10 nm and the error percentage is always <2%.
