# Supplementary material for: Optofluidic UV-Vis spectrophotometer for online monitoring of photocatalytic reactions
Source: Sci Rep. 2016 Jun 29;6:28928. doi: 10.1038/srep28928 (PMC4926220; doi:10.1038/srep28928)
Supplement: Supplementary Information [file srep28928-s1.doc]

**Optofluidic UV-Vis spectrophotometer for online monitoring of photocatalytic reactions**

Ning Wang*^ab^*, Furui Tan*^ab^*, Yu Zhao*^c^*, Chi Chung Tsoi*^a^**^b^*, Xudong Fan*^d^*, Weixing Yu*^e^* and Xuming Zhang**^ab^*

*^a^The Hong Kong Polytechnic University Shenzhen Research Institute, Shenzhen, P.R. China.*

*^b^Department of Applied Physics, The Hong Kong Polytechnic University, Hong Kong, P.R. China.*

*^c^Institute of Functional Nano & Soft Materials (FUNSOM) & Collaborative Innovation Center of Suzhou Nano Science and Technology, Jiangsu Key Laboratory for Carbon-Based Functional Materials & Devices, Soochow University, Suzhou, Jiangsu, P.R. China.*

*^d^Department of Biomedical Engineering, University of Michigan, Ann Arbor, MI 48109, USA.*

*^e^Key Laboratory of Spectral Imaging Technology, Xi'an Institute of Optics and Precision Mechanics, Chinese Academy of Sciences, Xi’an, Shaanxi, P.R. China.*

** E-mail: apzhang@polyu.edu.hk; Fax: +852 23337629; Tel: +852 34003258*

Supplementary information

**Fabrication of TiO_2_ porous film.** The sol-gel method was used for preparing porous TiO_2_ film [1]. First, 6 g TiO_2_ powders (Degussa P25, R:A= 3:7, 25 nm, 55 m2/g) was slowly added into 60 ml DI water, which contained acetylacetone (0.2 ml, Sigma-Aldrich) with stirring. After the powder was well dispersed, a detergent Triton X-100 (0.1 ml, Sigma) was added. Finally, 1.2 g polyethylene glycol (PEG20000, Sigma) was added into the solution under continuous stirring overnight. Secondly, painting method was chose for fabricating the film. A scotch tape (about 40 µm thick) mask was used to cover four edges of a glass slide and created a central hollow region of 1 cm × 1 cm, which is shown in Fig. S1. Then a layer of uniform TiO_2_ colloid was painted by a glass rod. After drying at 80 ℃, the scotch tapes were removed and the glass slide with film was annealled for 2 h at 500 ℃ in air. The film was observed using scanning electron microscope. The SEM photos of the film and its cross-section can be seen from our previous work [1]. It can be observed that the resultant TiO_2_ has sub-micron porous structures and good homogeneity. The film thickness is 2 µm.


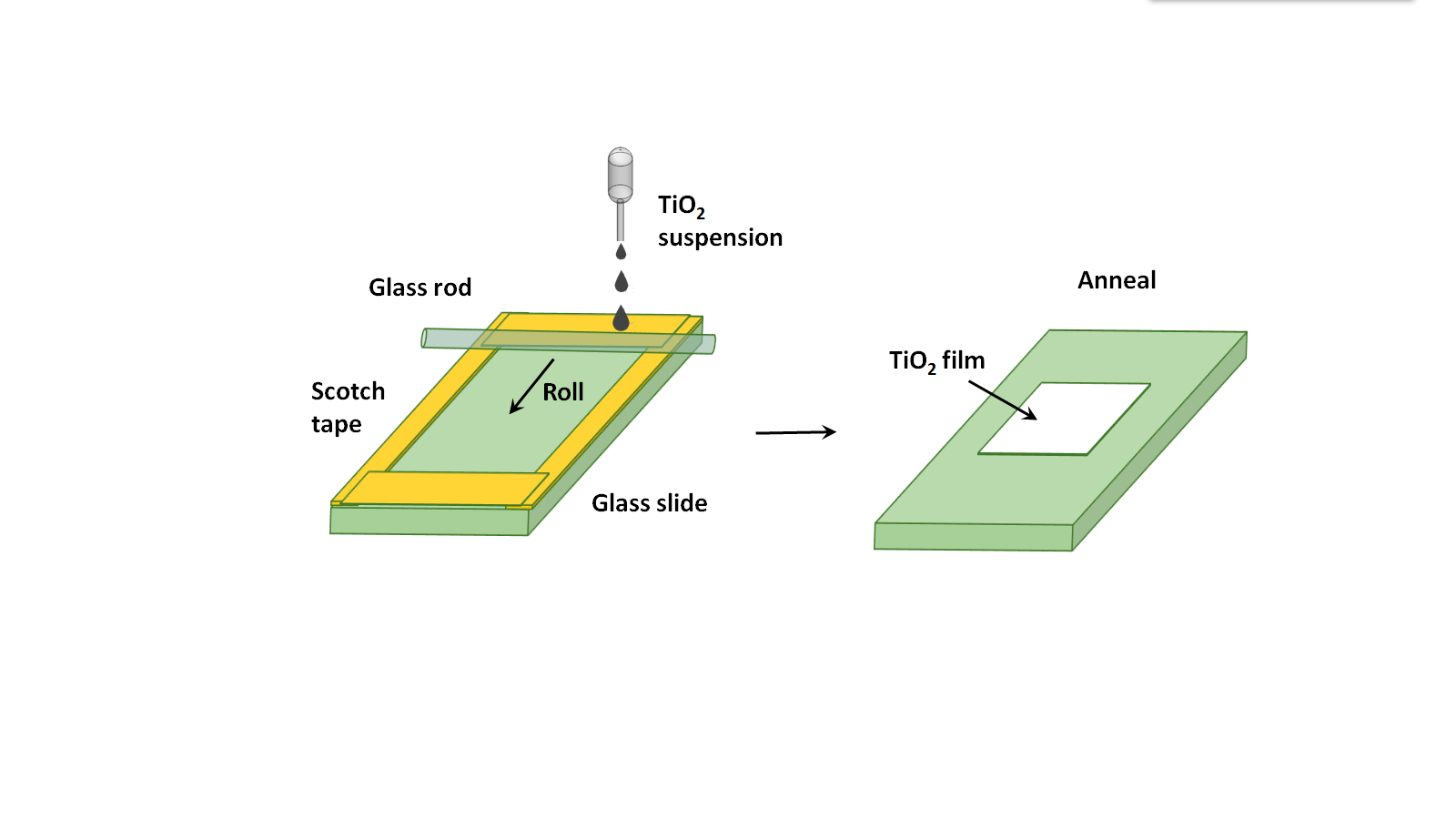


Figure S1 The schematic diagram of the painting method

**Fabrication of integrated device. When** fabricating the integrated device, standard UV photolithography was employed. However, the process of photolithography was taken twice here. First, a layer of SU-8 50 resin adhesive (Fig.S2(a)) was coated on a silicon wafer with spinning rate of 1200 rpm and exposed to UV light for 40 seconds with the photomask 1(Fig.S2(b)). After the removal of the unexposed area, the SU-8 mold was obtained on the silicon wafer, which including the reaction chamber and flow cells with the thickness of 100 µm (Fig.S2(c)). Second, after baking for several minutes, the other layer of SU-8 50 was spin coated onto the SU-8 mold 1 (Fig.S2(d)), which was then exposed to UV light for 40 seconds with the photomask 2(Fig.S2(e)) which only exposes the part of flow cells and form their thickness of 200 µm. The SU-8 mold 2 was formed (Fig.S2(f)), before the next step the quartz sheet were insert onto the mold which was described in text. Third, a prepolymer solution of PDMS in a 10:1 mixture ratio was poured on and cured at 70 ^o^C for 1 h. Finally, the cured transparent PMDS layer with all the microstructures was peeled off from the silicon wafer (Fig.S2(g)). Then the inlet and outlet ports are punched in the PDMS layer. In the last step, the chip was permanently bonded (Fig.S2(h)) again the TiO_2_ film coated glass slide after oxygen-plasma surface treatment.


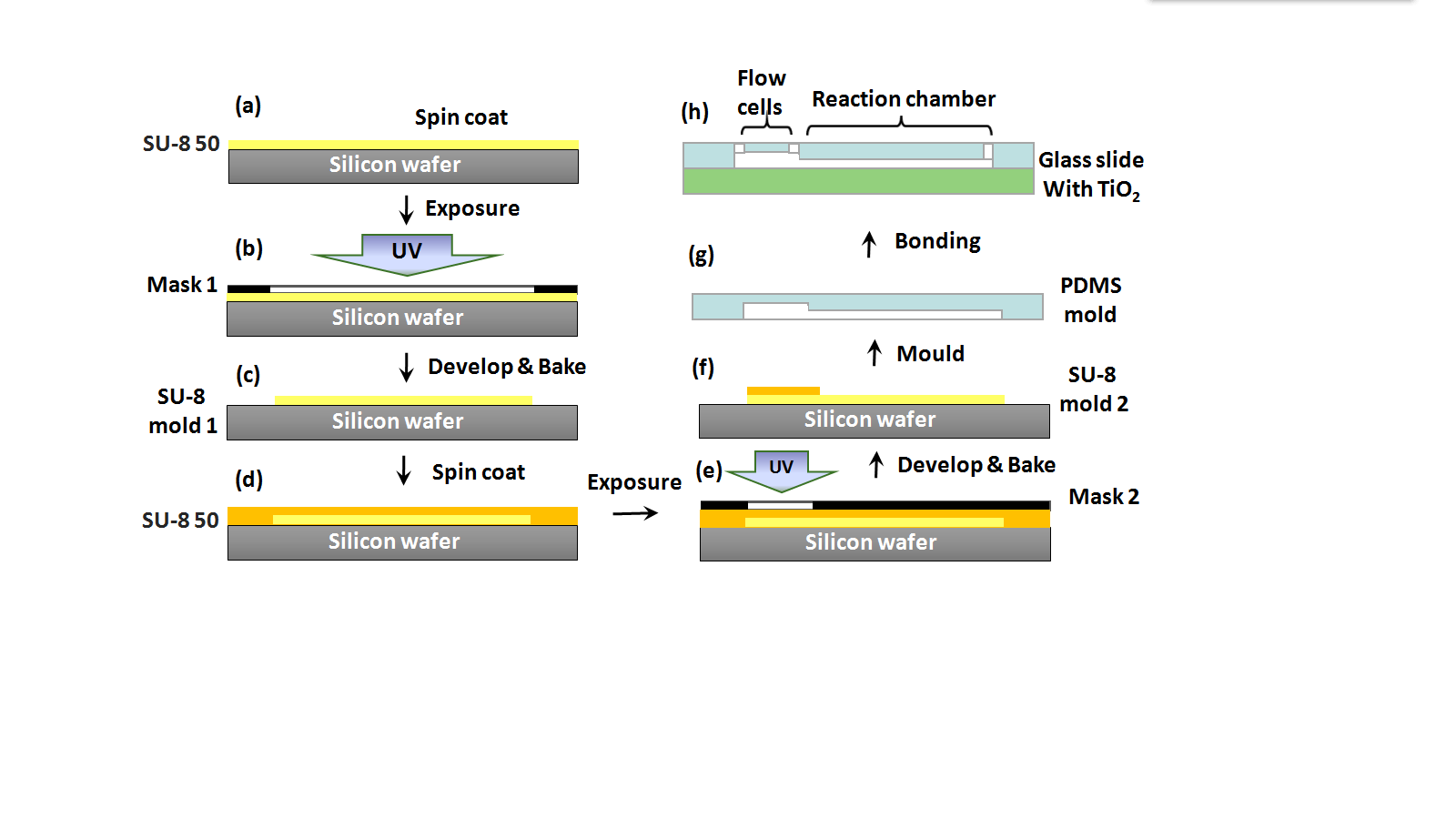


Figure S2 The process of fabricating the integrated device. (a) spin coat the first layer of SU-8 50 onto silicon wafer; (b) expose with the photomask 1 with structure of reaction chamber and flow cells; (c) develop and bake to form the first SU-8 mold; (d) spin coat the second layer of SU-8 50 onto the first layer with structures; (e) expose with the photomask 1 with structure of flow cells; (f) develop and bake to form the second SU-8 mold (height of reaction chamber : 100 µm, flow cells: 200µm); (g) mould the PDMS mold with all the structures; (h) bonding the PDMS mold onto the TiO_2_ film coated glass slide.

**Reference**

1. Lei, L. *et al.* Optofluidic planar reactors for photocatalytic water treatment using solar energy. *Biomicrofluidics* **4,** 43004 (2010).
